# Alzheimer's Disease Pathogenesis Perturbs Specific Spatial Protein Isoforms

**DOI:** 10.1002/alz70855_103003

**Published:** 2025-12-23

**Authors:** Daniel B McClatchy, Sergio R Labra, Christine Baal, Stuart A Lipton, John R Yates

**Affiliations:** ^1^ Scripps Research, La Jolla, CA, USA; ^2^ Scripps Research, La Jolla, CA, CA, USA

## Abstract

**Background:**

Unbiased mass spectrometry (MS) analyses have quantitated hundreds of Alzheimer's Disease (AD) human brain samples to reveal potential druggable pathways. Proteins exist as spatial isoforms with each localized to unique subcellular compartments. These studies, however, lack any spatial information about the AD perturbations. This study performed quantitative MS analysis on biological fractions on three different AD samples to determine the vulnerability of different spatial isoforms to pathogenesis.

**Method:**

The following samples were analyzed by quantitative MS: 1)27 human post‐mortem hippocampi from 13 AD and 14 ND (non‐demented) individuals, 2) Human cortical AD organoids with either PSEN1(WT/M146V) or APP(WT/swedish) mutations and isogenic control organoids cultured for 3 months, 3) Cortices from AD (APPswe/PSENdelta9) mice and wild‐type litter mates at 2, 5, and 12 months old. All samples were fractionated into 4 biological fractions. Each fraction was labeled with TMT (tandem mass tags) for quantification by an Orbitrap Tribrid mass spectrometer using SPS‐MS3 acquisition. Mice were fed azidohomoalanine (AHA) diet for 4 days, then sacrificed or returned to a normal diet for 7 days before sacrificed. This AHA pulse‐chase strategy allows for quantitation of protein degradation rates.

**Result:**

In the human hippocampi, fraction specific protein identifications revealed an enrichment of unique subcellular compartments. There were 1905 unique proteins observed to significantly different between AD and ND when the fraction MS datasets were combined. Seventy‐eight percent of these changes were specific to one fraction even though 89% of these proteins were quantified in more than one fraction. These protein perturbations were significantly enriched in the biological process of vesicle transport which included endosomal and nucleo‐cytoplasmic trafficking. Reciprocal expression patterns of identical proteins were quantitated in different fractions indicating protein mislocalization. Analysis of AD organoids replicated the findings of vesicle transport dysfunction. In the AD mouse model, global differences in protein degradation rates were observed between AD and WT as early as 2months, but the number of differences were dramatically increased with age. Identical proteins quantitated in different fractions possess distinguishable degradation rates.

**Conclusion:**

AD pathogenesis perturbs specific protein isoforms which may stem from the dysfunction of protein trafficking or local degradation pathways.

